# Diagnostic Performance of Simultaneous [^18^F]-FDG PET/MR for Assessing Endoscopically Active Inflammation in Patients with Ulcerative Colitis: A Prospective Study

**DOI:** 10.3390/jcm9082474

**Published:** 2020-08-01

**Authors:** Jost Langhorst, Lale Umutlu, Benedikt Michael Schaarschmidt, Johannes Grueneisen, Aydin Demircioglu, Michael Forsting, Karsten Beiderwellen, Johannes Haubold, Jens Matthias Theysohn, Anna Katharina Koch, Gustav Dobos, Alexander Dechêne, Ken Herrmann, Nils Martin Bruckmann, Thomas Lauenstein, Yan Li

**Affiliations:** 1 Department for Internal and Integrative Medicine, Sozialstiftung Bamberg, Klinikum am Bruderwald, Buger Straße 80, 96049 Bamberg, Germany; j.langhorst@kem-med.com; 2 Department of Internal and Integrative Medicine, University of Duisburg-Essen, Kliniken-Essen-Mitte, Am Deimelsberg 34 a, 45276 Essen, Germany; a.koch@kem-med.com (A.K.K.); g.dobos@kem-med.com (G.D.); 3 Institute of Diagnostic and Interventional Radiology and Neuroradiology, University Hospital Essen, Hufelandstraße 55, 45147 Essen, Germany; lale.umutlu@uk-essen.de (L.U.); benedikt.schaarschmidt@uk-essen.de (B.M.S.); Johannes.grueneisen@uk-essen.de (J.G.); aydin.demircioglu@uk-essen.de (A.D.); michael.forsting@uk-essen.de (M.F.); karsten.beiderwellen@uni-due.de (K.B.); johannes.haubold@uk-essen.de (J.H.); Jens.theysohn@uk-essen.de (J.M.T.); 4 Department of Gastroenterology, Hepatology and Endocrinology, General Hospital Nuremberg, Prof.-Ernst-Nathan-Str. 1, 90419 Nürnberg, Germany; alexander.dechene@klinikum-nuernberg.de; 5 Department of Nuclear Medicine, University Hospital Essen, Hufelandstraße 55, 45147 Essen, Germany; ken.herrmann@uk-essen.de; 6 Institute of Diagnostic and Interventional Radiology and Neuroradiology, University Hospital Düsseldorf, Moorenstr. 5, 40225 Düsseldorf, Germany; Nils-Martin.Bruckmann@med.uni-duesseldorf.de; 7 Department of Radiology, Evangelisches Krankenhaus Düsseldorf, Kirchfeldstraße 40, 40217 Düsseldorf, Germany; thomas.lauenstein@evk-duesseldorf.de

**Keywords:** ulcerative colitis, diagnosis, positron-emission tomography, inflammatory bowel diseases, PET, MR enterography

## Abstract

**Background:** To investigate the diagnostic performance of simultaneous ^18^F-fluoro-deoxyglucose ([^18^F]-FDG) PET/MR enterography in assessing and grading endoscopically active inflammation in patients with ulcerative colitis. **Methods:** 50 patients underwent PET/MR 24 h before ileocolonoscopy. Inflammatory activities of bowel segments were evaluated with both Mayo endoscopic subscore and Nancy histologic index. MR, DWI (Diffusion-weighted imaging) and PET were utilized as qualitative parameters for detecting endoscopically active inflammation. SUVmaxQuot in each segment (maximum of standard uptake value relative to liver) was calculated to quantify inflammation. **Results:** In the study arm without bowel purgation, combined reading of PET and MR resulted in significantly increased specificity against each submodality alone (0.944 vs. 0.82 for MR and 0.843 for PET, *p* < 0.05) and highest overall accuracy. In the study arm with bowel purgation, the significantly lower specificity of PET (0.595) could be markedly improved by a combined reading of PET and MR. Metabolic conditions in bowel segments with both endoscopic and histological remission were significantly lower than in segments with endoscopic remission but persistent microscopic inflammation (SUVmaxQuot 0.719 vs. 0.947, *p* < 0.001). SUVmaxQuot correlated highly with Mayo endoscopic subscore (ρ = 0.718 and 0.606) and enabled grading of inflammatory activity. **Conclusions:** Simultaneous [^18^F]-FDG PET/MR may be considered as an alternative to endoscopy in clinical trials.

## 1. Introduction

Ulcerative colitis (UC) is a chronic inflammatory bowel disease (IBD) with relapsing–remitting courses. According to expert consensus-based recommendation, the selected treatment-target for UC was clinical and endoscopic remission (Mayo endoscopic subscore [MES] of 0–1) [1]. Accumulating data indicated the importance of including histological remission into therapeutic endpoint, since histological remission might better predict lower rates of corticosteroid use and acute severe colitis requiring hospitalization [2]. Persistence of microscopic inflammation in spite of macroscopically inactive disease or clinical remission was significantly associated with the risk of clinical relapses [3,4]. Therefore, a composite of endoscopic and histological mucosal healing toward complete remission might be favored in clinical trials and practice. 

However, there are several drawbacks associated with colonoscopy, including procedural discomfort, the invasive nature and low patient acceptance [5,6]. Hence, reliable and non-invasive diagnostic modalities are desirable. Recently, superior diagnostic performance of simultaneous ^18^F-fluoro-deoxyglucose ([^18^F]-FDG) Positron Emission Tomography (PET)/MR have been demonstrated in the assessment of IBD [7,8,9,10]. Hybrid biomarkers comprised of PET and MR parameters facilitated higher diagnostic values than each modality alone not only in assessing disease activity but also in differentiating fibrotic strictures from mixed or inflammatory ones in Crohn’s disease [11,12]. Previously, in a randomized controlled trial (RCT), we have investigated the diagnostic accuracies of [^18^F]-FDG PET/MR in predicting histologically active inflammation in 50 patients with UC [13]. Our results revealed the strong association between the change of metabolic condition of bowel wall and the degree of neutrophil infiltrate.

Given the fact that endoscopic remission is still considered as therapeutic endpoint and mismatch between endoscopic and histologic findings is common especially in endoscopically inactive or mild disease [3,14], we performed a second analysis of our RCT to evaluate the diagnostic performance of PET/MR in assessing endoscopically active inflammation. Furthermore, we aimed to define and compare the optimal cutoff-values for PET parameter in predicting endoscopic remission and complete remission. Finally, extra-intestinal findings regarding their IBD specificity were evaluated.

## 2. Materials and Methods

### 2.1. Population and Study Design

This study is part of an RCT (clinicaltrials.gov NCT03781284). Between November 2015 and April 2017, 50 UC Patients who required endoscopic assessment because of clinical symptoms of flares or follow-up as disease control were recruited. The study was approved by the ethics committee of the University of Duisburg-Essen (number 11-4824-BO) and conducted in accordance with the declaration of Helsinki. Written informed consent was obtained from all participants.

Inclusion criteria included age older than 18 years, confirmed diagnosis of UC (based on defining symptoms, endoscopic and histopathologic findings) and clinical indication to undergo ileocolonoscopy.

Exclusion criteria were Pregnancy, MR contraindications (e.g., cardiac pacemaker or neurostimulation system), severe claustrophobia, severe renal failure (glomerular filtration rate <30 mL/min) and diabetes under continuous medication of metformin.

### 2.2. Randomization and Masking

**Figure 1:**
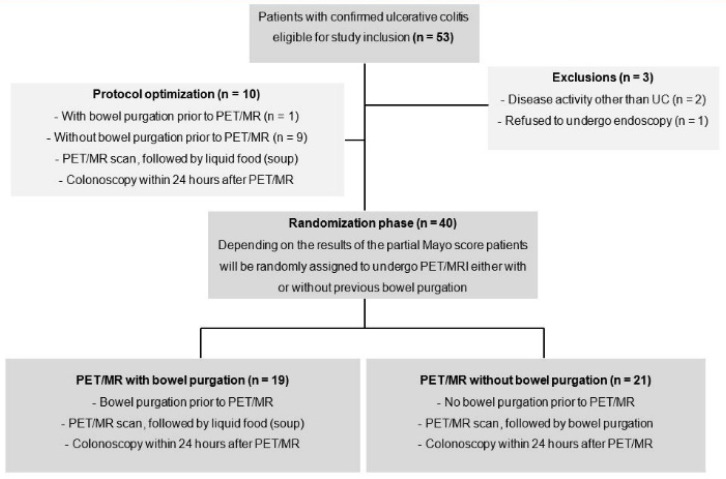
Study flowchart.

After an initial study phase with 10 non-randomized patients (nine without and one with previous bowel purgation) for protocol optimization, 40 patients were randomly assigned to one of the two study arms with or without bowel purgation before PET/MR. Randomization was performed software-based (TenAlea) by an independent institution (Center for Clinical Trials Essen) using one stratification factor and an allocation ratio of 1:1. The stratification factor was determined by clinical disease activity: symptomatic disease or remission (partial Mayo score >1 or ≤1). Depending on randomization, bowel purgation took place before or after PET/MR examination (Figure 1).

### 2.3. Endoscopic and Histologic Assessment

For bowel purgation, 3000 mL of an electrolyte solution (Polyethylene Glycol, Braintree Laboratories) was ingested the evening before either ileocolonoscopy or PET/MR depending on randomization. To avoid artificial post-biopsic mucosal FDG-uptake, ileocolonoscopies were completed after PET/MR within 24 h. Ileocolonoscopy was performed by a board-certified gastroenterologist and reviewed by a second gastroenterologist, who were blinded by PET/MR results. From rectum to terminal ileum, seven ileocolonic segments (rectum, sigmoid colon, descending colon, transverse colon, ascending colon, cecum and terminal ileum) were divided (Supplementary 1). MES for each segment was calculated independently and discrepancy was resolved by consensus. Endoscopically active inflammation was defined as MES ≥ 1 and inactive disease as MES = 0. The histological activity was evaluated with Nancy index (Supplementary 2) [15]. Histologically active inflammation was defined as Nancy index >1 and quiescent disease as ≤1. A composite of endoscopic and histologic remission defined complete remission.

### 2.4. [^18^F]-FDG-PET/MR Enterography Protocol and Imaging Analysis

The imaging was performed by using a 3.0 tesla whole-body PET/MR (Biograph mMR, Siemens Healthcare, Erlangen, Germany). The dedicated imaging protocol including patient’s preparation was summarized in Supplementary Material (Supplementary 3).

A post-processing software (Syngo.via, VB30B, Siemens Healthcare) was used for imaging analysis. As quantitative PET parameter, SUVmaxQuot was calculated as the ratio of SUVmax of each bowel segment relative to SUVmax of liver [16]. The SUVmax of bowel segment was measured by placing a spherical volume of interest in the most FDG-avid part (mean size 9 ± 3.6 cm^3^) and the SUVmax of liver was calculated by the same way using a larger spherical volume (mean size 50.2 ± 20.4 cm^3^). If SUVmaxQuot was ≥1, PET was considered positive for active disease [17,18]. For MR imaging, bowel segment was considered positive, if all following criteria were fulfilled: [19,20] (1) presence of hyperintense mucosa in the contrast-enhanced, fat-suppressed and T1-weighted imaging; (2) positive comb-sign indicating engorged vasa recta and hyperemia; (3) thickening of colonic wall, even if it was only slightly thickened compared to ileal segements. Diffusion-weighted imaging (DWI) was evaluated separately and considered positive, if hyperintense bowel wall could be observed in the *b*1000 s/mm^2^ of DWI. Imaging was evaluated independently by two radiologists (each with 4 years hybrid-imaging experiences), who were blinded to the endoscopic results. In addition, extraintestinal findings were evaluated regarding their IBD specificity and whether further work-up was necessary.

### 2.5. Statistical Analysis

The two study arms regarding sociodemographic and clinical characteristics were compared with each other using Student’s *t*-tests for continuous and chi-square tests for categorical data. To test the diagnostic accuracies of categorical parameters (PET, MR and DWI) against ileocolonoscopy, data were evaluated on segment basis using chi-square tests. The sensitivities, specificities and accuracies of categorical PET/MR parameters within the study arm were compared against each other using McNemar tests. Correlation between SUVmaxQuot and MES was calculated with Spearman’s rank correlation test. Receiver operating characteristics (ROC) curves were made and area under the curves (AUC) was calculated for SUVmaxQuot. Optimized cut-off points were determined by the maximum of Youden’s indices. Mann–Whitney *U* test was run to compare median values of continuous parameters. Extraintestinal findings were analyzed descriptively. 

All analyses were performed with the Statistical Package for Social Sciences software (SPSS 22.0; IBM). *p*-values for multiple testing was adjusted using Holm′s method. A *p*-value <0.05 was considered significant.

## 3. Results

### 3.1. Patient’s Characteristics

Fifty-three patients were enrolled in the study. One patient who refused to undergo endoscopy and other two patients with disease activity others than UC were excluded from the study. Patients’ sociodemographic characteristics were shown in Table 1. There were no significant baseline differences between the study arms (all *p* > 0.05). One-hundred and forty-seven bowel segments of 21 patients without bowel purgation prior to PET/MR could be evaluated both with PET/MR and ileocolonoscopy. According to endoscopy, active inflammation was present in 58 segments (mild colitis *n* = 23, moderate *n* = 20, severe *n* = 15). For another 19 patients with bowel purgation, 131 bowel segments could be analyzed because of failed intubation of terminal ileum in two patients. Active inflammation could be found in 57 segments (mild colitis *n* = 9, moderate *n* = 28, severe *n* = 20).

### 3.2. Diagnostic Performance of [^18^F]-FDG-PET/MR Enterography in Study Arm Without Bowel Purgation

In detecting endoscopically active inflammation, MR, DWI and PET performed similarly well in specificity and overall diagnostic accuracy (Table 2); however, the sensitivity of DWI was considerably lower than contrast-enhanced T1-weighted MR and PET. Nevertheless, no significant difference regarding McNemar Test could be found between MR, DWI and PET. Discrepant results of MR and PET (*n* = 13 MR was positive and PET was negative; *n* = 10 MR was negative and PET was positive) could be found in 23 segments, in which 20 segments showed normal finding in the endoscopy. Therefore, it could be proposed that PET-MR, as combined reading of both MR and PET, was considered positive only if both MR (morphological criteria) and PET (SUVmaxQuot ≥ 1) were positive. In other cases (discrepancy occurred or both parameters were negative), PET-MR was considered negative. 

The newly proposed PET-MR resulted in a significantly increased specificity of 0.944 compared to each sub-modality alone (vs. 0.82 for MR and 0.843 for PET, both *p* < 0.05) and only a slightly reduced sensitivity of 0.862. Furthermore, PET-MR led to the highest overall diagnostic accuracy compared to other 3 PET/MR parameters.

### 3.3. Diagnostic Performance of [^18^F]-FDG-PET/MR Enterography in Study Arm with Bowel Purgation

No significant difference regarding sensitivity was found between MR, DWI and PET (Table 2). However, the specificity of PET was significantly lower than MR and DWI (0.595 vs. 0.875 and 0.811, *p* < 0.01). In 31 bowel segments with discrepant MR and PET results (*n* = 5 MR was positive and PET was negative; *n* = 26 MR was negative and PET was positive), 23 segments showed normal endoscopic findings. The aforementioned PET-MR again resulted in highest specificity among all PET/MR parameters. Due to its lower specificity, PET alone showed significantly reduced diagnostic accuracy against MR, DWI or PET-MR (all *p* < 0.05). MR resulted in the highest diagnostic accuracy.

### 3.4. Grading of Endoscopic Disease Activity with SUVmaxQuot

According to ROC for detecting endoscopically active inflammation, the diagnostic performance of SUVmaxQuot was better in study arm without bowel purgation (AUC = 0.921 vs. 0.836, Table 3 and Supplementary 4). Correspondingly, the sensitivity and specificity of SUVmaxQuot was also higher in those without bowel purgation (0.879 and 0.843 vs. 0.768 and 0.838). SUVmaxQuot correlated highly with MES according to Spearman’s rank correlation test and the correlation was higher in study arm without bowel purgation (ρ = 0.718 vs. 0.606, both *p* < 0.001). However, for detecting endoscopically severe inflammation (MES = 3), AUC of ROC for the SUVmaxQuot was higher in study arm with purgation (0.863 vs. 0.816).

### 3.5. Predicting Endoscopic Remission and Complete Remission with SUVmaxQuot

In total, 317 out of 350 bowel segments could be assessed both endoscopically and histologically because of histological sampling error and failed intubation of terminal ileum. Microscopic inflammation was found in 55 (44 mild, 8 moderate to severe, 3 ulcerative inflammation) of the 180 bowel segments with endoscopic remission. The median value for SUVmaxQuot in bowel segments with endoscopic remission but histological active inflammation was significantly higher than in bowel segments with complete remission (0.947 vs. 0.719, *p* < 0.001, Mann–Whitney *U* test). Of the 140 bowel segments with histological remission, 15 showed endoscopic active inflammation and the rest showed complete remission. For detecting endoscopic remission, an optimal cutoff-value of 1.29 for SUVmaxQuot resulted in a sensitivity of 0.719, specificity of 0.9 and accuracy of 0.828. For predicting complete remission, the optimal cutoff value of 0.99 was markedly lower with comparable diagnostic performance (sensitivity 0.74, specificity 0.84 and accuracy 0.831).

### 3.6. Extra-Intestinal Findings

Extra-intestinal findings could be found in 17 patients. Three IBD-specific findings were primary sclerosing cholangitis (PSC), inactive sacroiliitis and perianal fistula. Five IBD non-specific findings that needed further clinical investigations were inguinal lymphadenopathy, adenoma of the right adrenal gland, granuloma in the lung, pathologic tracer uptake in the endometrium uteri and subchondral cyst-like lesion in the acetabulum. Another nine IBD non-specific findings that did not require follow-up included splenic cyst, cholecystolithiasis, two hemangioma in the liver, liver cysts, hiatal hernia, kidney cysts, fracture of lumbar vertebral body.

## 4. Discussion

Evaluation of disease activity and extent is of crucial importance for clinicians in choosing the proper treatment. In acute attack of UC, ileocolonoscopy is associated with increased rate of complications. The alternative diagnostic test should be non-invasive, easy to perform and correlate well with disease activity. Our study aimed to investigate the superior diagnostic value of [^18^F]-FDG PET/MR enterography in detecting endoscopically active inflammation and predicting complete (endoscopic and histologic) remission in patients with UC. Our study included three key findings that we considered important. First, [^18^F]-FDG PET/MR enabled detection of endoscopically active inflammation and grading of disease activity with high diagnostic accuracy. Second, the newly proposed PET-MR as combined reading of both PET and MR parameters resulted in the highest diagnostic specificity regardless of bowel cleansing. Third, metabolic conditions in bowel segments with complete remission was significantly lower than in segments with only endoscopic remission but persistent microscopic inflammation. Therefore, SUVmaxQuot facilitated prediction of endoscopic remission and complete remission with different cutoff values. 

**Figure 2:**
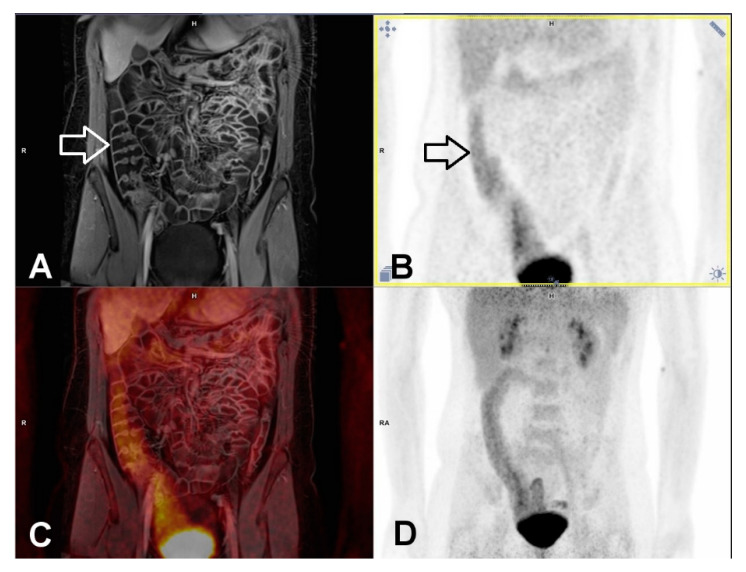
False-positive PET finding in ascending colon in a 29-year-old female patient with previous bowel purgation. Intensive tracer uptake (above liver level) could be clearly demonstrated in ascending colon and adjacent terminal ileum (black arrow in B), though active inflammation was found neither in MR (white arrow in A) nor by colonoscopy. Histology confirmed the absence of neutrophil infiltrate. The combined reading of PET-MR can obviate false-positive interpretation of PET. Notice the good distension of ascending colon following bowel cleansing and oral intake of fluid. (**A**) MR imaging. T1w-3D-VIBE with fat-saturation in the portalvenous phase, (**B**) PET, (**C**) fused imaging of PET and MR, (**D**) maximum intensity projection of PET.

For an optimal morphological evaluation with MR imaging, a well-distended bowel segment without residual feces is preferred. MR enterography with bowel purgation might fulfill this condition. Indeed, in detecting endoscopically active inflammation MR showed the highest diagnostic accuracy in study arm with previous bowel cleansing. However, taking SUVmaxQuot ≥ 1 as cutoff [17], PET component resulted in a strikingly lower specificity, which might be explained by the generally increased FDG-uptake in the smooth muscle and mucosa layer of colonic segments and increased FDG-secretion into the bowel lumen due to bowel preparation (Figure 2) [21,22]. After purgation, no inflammatory cell infiltrate such as neutrophils were present in bowel segments under histologic remission. Therefore, the bowel preparation might result in false-positive interpretation of PET [13]. As demonstrated in our study, such misinterpretation of PET could be avoided by combined reading of PET and MR.

**Figure 3:**
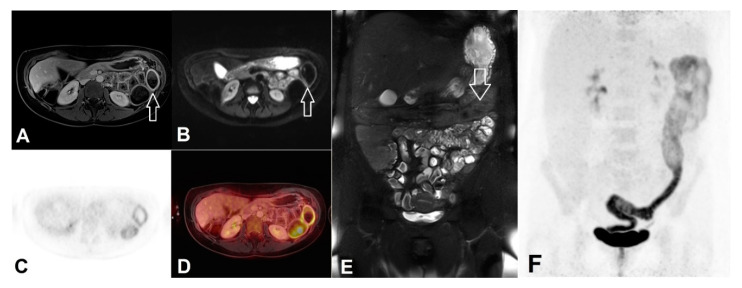
Left-sided colitis in a female patient without previous bowel purgation. Despite feces in colon, left-sided colitis could be found in all PET/MR modalities as well in the ileocolonoscopy. (**A**) Intensive mucosal enhancement of contrast agent in left colon (white arrow) in T1w-3D-VIBE with fat saturation, (**B**) Hyperintensity of bowel wall shown in the *b*1000 s/mm^2^ of DWI, (**C**) PET, (**D**) Fused imaging of PET and MR, (**E**) Feces in the left-sided colon (white arrow) shown in the fat-saturated T2w HAST (**F**) Maximum intensity projection of PET demonstrating increased tracer uptake throughout the left-sided colon.

Other than CD, in which wall thickness was considered the most important diagnostic parameter for inflammation detection [8], inflamed colonic segments in UC showed only mild wall thickening. Residual feces in uncleansed bowel segments did not obviate evaluation of other imaging signs of inflammation such as comb sign or hyperintense mucosa layer induced by hyper-enhancement of contrast agent (Figure 3). That might explain why the overall accuracy of MR was only slightly improved with the help of previous bowel cleansing (0.88 vs. 0.85). In a study investigating DWI-MR colonography in 35 UC patients without bowel preparation [23], hyper-enhancement of mucosal layer alone demonstrated superb sensitivity (0.882) and specificity (0.833) in detecting endoscopically active inflammation, which could be reproduced in our study (vs. 0.897 and 0.82). The sensitivity for DWI in study arm without purgation was noticeably lower than their result (0.78 vs. 0.91) possibly due to higher *b* value of DWI and stronger magnetic field used in our study (1000 vs. 600 s/mm^2^ and 3 vs. 1.5 tesla). It could be demonstrated in a research with the same study design that DWI hyperintensity at *b* = 800 s/mm^2^ led to significantly greater diagnostic accuracy than at b = 400, 600 and 1000 s/mm [24]. It is of note that the overall diagnostic accuracy of DWI was not inferior without bowel purgation (0.837 vs. 0.835). Under uncleansed bowel condition, the aforementioned combined reading of PET-MR exhibited even the highest diagnostic specificity and overall accuracy.

Our study revealed that simultaneous [^18^F]-FDG PET/MR enterography not only facilitated detection of endoscopically active inflammation but also enabled grading of inflammatory activity by means of SUVmaxQuot, which correlated highly with MES (ρ = 0.718 and 0.606, both *p* < 0.05). Based on higher cutoff values, SUVmaxQuot might predict bowel segments with severe ulcerative inflammation (MES 3). This result was supported by a former study with 15 UC patients utilizing PET/CT, which demonstrated significantly increased FDG-uptake (SUVmaxQuot between 1 and 3) in segments with endoscopically severe inflammation (spontaneous bleeding, severe friability and/or ulcerations) [17]. In addition, SUVmaxQuot is a reliable quantitative parameter with excellent inter-observer concordant measurement [13]. 

Until now, endoscopic assessment remains the gold standard to evaluate the inflammatory activity of UC, although results of previous studies indicated the need of including histological remission into therapeutic endpoint. In line with previous findings [3,4,14], histologically active inflammation could be found in about 30% of bowel segments with endoscopic remission in our study. Our results confirmed that complete remission was distinct from endoscopic mucosal healing in UC, since the metabolic condition of bowel segments with complete remission was significantly lower than bowel segments with only endoscopic remission but persistent histological inflammation (median value of SUVmaxQuot 0.947 vs. 0.719, *p* < 0.001). With lower cutoff value, SUVmaxQuot provided the possibility of differentiating the bowel segments with complete remission from those with only endoscopic remission. 

**Figure 4:**
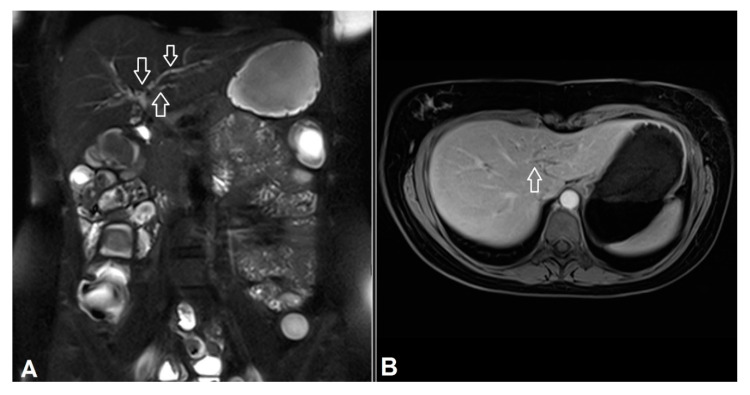
Primary sclerosing cholangitis in a 25-year-old female patient. The intrahepatic bile ducts were irregularly configured with multiple strictures (white arrows in A) and dilatation (white arrow in B). (**A**) Fat-saturated T2w HASTE. (**B**) T1w-3D-VIBE with contrast agent and fat saturation.

Compared to ileocolonoscopy, one advantage of PET/MR was the possibility to assess extra-intestinal manifestations of IBD and MR imaging hereby plays a key role. Pathognomonic MR morphological signs of PSC were found in one young female in our study (Figure 4). This finding is clinically relevant, since UC patients with PSC showed increased risk of cholangiocarcinoma and were more likely to have more extensive UC than those without PSC [25,26]. Another advantage of PET/MR especially the PET component was its simplicity and objectivity to measure and to interpret with less inter-observer variability. With additional MR parameter, PET-MR as combined reading allowed reduced false-positive interpretation and improved diagnostic confidences. However, the cramped and uncomfortable prone position over long examination time and loud background noise as well as the associated radiation exposure limited patient’s acceptance for PET/MR [27], besides its limited availability and higher costs. Therefore, PET/MR might be considered only in patients with suspected active disease suggested by various clinical or lab chemical parameters such as fecal biomarkers [28].

Our study has several limitations. First, it is a one-center observational study with limited participants. The subdivision of the overall population into different subsets might dilute the statistical effect. Second, the oral intake of large amount of fluid might affect physiological bowel motility, which could negatively influence the FDG-uptake level of bowels. This aspect should be further investigated in a study without oral intake of fluid or bowel preparation. Third, though there is no significant difference regarding clinical baselines between both study arms, in the study arm with bowel purgation the percentile of endoscopic severe inflammation was higher (*n* = 20 out of 57 vs. *n* = 15 out of 58). This difference might be the reason of greater AUC of ROC for PET in detecting severe inflammation, though the diagnostic performance of PET in study arm without bowel preparation was expected to be higher.

## 5. Conclusions

Simultaneous [^18^F]-FDG PET/MR may serve as a non-invasive alternative to endoscopy without the need of bowel purgation. For detecting endoscopically active inflammation, PET-MR as combined reading of PET and MR provides higher diagnostic accuracy than each sub-modality alone. With lower cutoff-value, SUVmaxQuot facilitated to differentiate bowel segments with complete remission from those with endoscopic remission.

## Figures and Tables

**Table 1:** Sociodemographic and clinical characteristics (mean ± standard deviation (range)).

	Total (*n* = 40)	With Bowel Purgation (*n* = 19)	Without Bowel Purgation (*n* = 21)	*p* Values
Age years	42.8 ± 12.1 (23–66)	42.4 ± 11.71 (23–63)	43.2 ± 12.7 (24–66)	0.63
Sex *n* (%)				0.11
Female	25 (62.5)	11 (57.9)	14 (66.7)	
Male	15 (37.5)	8 (42.1)	7 (33.3)	
Anamnestic pattern *n* (%)				0.2
Proctitis	8 (20)	3 (15.8)	5 (23.8)	
Left-sided colitis	16 (40)	9 (47.4)	7 (33.3)	
Pancolitis	16 (40)	7 (36.8)	9 (42.9)	
Time since diagnosis, years	12.65 ± 9.70 (1–42)	13.42 ± 10.48 (2–42)	11.95 ± 9.14 (1–42)	0.91
Smokers *n* (%)	2 (5)	0 (0)	2 (9.6)	n.a
Full Mayo Score	6.25 ± 2.91 (0–11)	6.68 ± 2.73 (1–11)	5.86 ± 3.07 (0–10)	0.4
Inactive *n* (%)	6 (15)	2 (10.5)	4 (19)	
Mild *n* (%)	6 (15)	3 (15.8)	3 (14.3)	
Moderate *n* (%)	27 (67.5)	13 (68.4)	14 (66.7)	
Severe *n* (%)	1 (2.5)	1 (5.3)	0 (0)	
SUVmaxQuot	2.92 ± 1.64 (0.82–8.28)	3.01 ± 1.40 (0.82–6.42)	2.83 ± 1.86 (0.88–8.28)	0.6
Blood values				
CRP	1.15 ± 1.28 (0–4.8)	1.43 ± 1.47 (0–4.8)	0.89 ± 1.04 (0–4.2)	0.16
ESR	19.00 ± 16.53 (2–70)	21.84 ± 20.26 (2–70)	16.43 ± 12.21 (2–47)	0.61
Leucocytes	8307.58 ± 3314.78 (874–14,040)	9234.74 ± 3636.98 (4470–18,330)	7468.71 ± 2822.37 (874–14,040)	0.12
Thrombocytes	338.88 ± 106.37 (135–580)	333.11 ± 102.61 (135–555)	344.10 ± 111.92 (217–580)	0.4
Medication *n* (%)				
Steroids	19 (47.5)	10 (52.6)	9 (42.9)	0.54
ThiopurineMTX	4 (10)	2 (10.5)	2 (9.5)	n.a
Biologics	6 (15)	5 (26.3)	1 (4.8)	n.a
Mesalamine	33 (82.5)	16 (84.2)	17 (81.0)	0.79
Other	22 (55)	9 (47.4)	13 (61.9)	0.36

Note. SUVmaxQuot = maximum of standardized uptake value ratio gut/liver; CRP = C-reactive protein; ESR = erythrocyte sedimentation rate; MTX = methotrexate; n.s. = not significant; n.a. = not available.

**Table 2:** Diagnostic performance of [^18^F]-FDG-PET/MR enterography in study arms with and without bowel purgation.

PET/MR Parameters	Study Arm without Bowel Purgation (*n* = 147 Segments)			Study Arm with Bowel Purgation (*n* = 131 Segments)		
	Sensitivity	Specificity	Accuracy	Sensitivity	Specificity	Accuracy
MR	0.897	0.820	0.850	0.877	0.875	0.880
DWI	0.776	0.876	0.837	0.860	0.811	0.835
PET	0.879	0.843	0.857	0.877	0.595	0.714
PET-MR	0.862	0.944	0.912	0.807	0.892	0.857
	***p*-Values in McNemar Test for Comparison between PET/MR Parameters**					
MR vs. DWI	0.096	0.681	1	1	0.454	0.54
MR vs. PET	1	1	1	1	<0.001	0.001
MR vs. PET-MR	1	0.005	0.105	0.75	1	0.75
DWI vs. PET	0.35	1	1	1	0.006	0.024
DWI vs. PET-MR	0.50	0.436	0.078	1	0.146	0.75
PET vs. PET-MR	1	0.024	0.105	0.75	<0.001	0.006

Note. MR = magnetic resonance; PET = positron emission tomography; DWI = Diffusion Weighted Imaging. *p*-values were adjusted using Holm’s method.

**Table 3:** Diagnostic performance of SUVmaxQuot in detecting endoscopically active and severe inflammation with and without purgatives.

	AUC of ROC for SUVmaxQuot	Sensitivity	Specificity	Optimal Cutoffs
**Diagnostic Performance of SUVmaxQuot in Detecting Active Inflammation (MES ≥ 1)**				
without bowel purgation	0.921	0.879	0.843	1.01
with bowel purgation	0.836	0.768	0.838	1.28
**Diagnostic Performance of SUVmaxQuot in Detecting Severe Inflammation (MES = 3)**				
without bowel purgation	0.816	0.867	0.727	1.47
with bowel purgation	0.863	0.842	0.829	1.77

Note. AUC = Area under the curve; ROC = receiver operating characteristics; MES = Mayo endoscopic subscore.

## Supplementary Materials

The following are available online at https://www.mdpi.com/2077-0383/9/8/2474/s1, Supplementary 1: Division of ileocolonic segments and grading of Mayo endoscopic subscore, Supplementary 2: Grading of Nancy Index, Supplementary 3: Imaging Protocol of PET-MR Enterography, Supplementary 4: Receiver operating characteristics curves of SUVmaxQuot in detecting endoscopically active and severe inflammation with and without purgatives.
