# Supplementary material for: Diagnostic Performance of Simultaneous [18F]-FDG PET/MR for Assessing Endoscopically Active Inflammation in Patients with Ulcerative Colitis: A Prospective Study
Source: J Clin Med. 2020 Aug 1;9(8):2474. doi: 10.3390/jcm9082474 (PMC7465255; doi:10.3390/jcm9082474)
Supplement: Supplementary file 1 [file jcm-09-02474-s001.pdf]

Supplemental material

Title: Diagnostic performance of simultaneous [^{18}F]-FDG PET/MR for assessing endoscopically active inflammation in patients with ulcerative colitis: a prospective study.

1. Division of ileocolonic segments and grading of Mayo endoscopic subscore

To allow comparability of colonoscopy procedures, we propose a definition of 7 ileocolonic segments based on landmarks: (1) terminal ileum: proximal of the ileocecal valve; (2) cecum: ileocecal valve to 15 cm distant from the ileocecal valve; (3) ascending colon: 30 cm distant from the ileocecal valve to the hepatic flecture; (4) transverse colon: hepatic flecture to splenic flecture; (5) descending colon: splenic flecture to 35 cm proximal to the anal verge; (6) sigmoid: 35 to 15 cm proximal to the anal verge; (7) rectum: 15 to 0 cm proximal of the anal verge.

Two independent gastroenterologists blinded to the PET/MR results calculated the Mayo endoscopic subscore with inactive (0) showing a normal mucosal appearance, mild (1) showing erythema, a decreased vascular pattern, minimal granularity, moderate (2) showing marked erythema, friability, granularity, absent vascular pattern, bleeding on minimal trauma, no ulceration and severe (3) showing ulceration and spontaneous bleeding. Mayo endoscopic subscore was calculated based on the seven-segment model for every segment.

2. Grading of Nancy Index

Three mucosal biopsies in each segment in the endoscopically most inflamed areas or randomly by absence of inflammation were obtained. The histological disease activity was judged with Nancy index. The grade 4 was defined as the presence of ulceration. When no ulceration was observed, grade 3 was defined if moderate to severe infiltrate of acute inflammatory cells were present. If only mild infiltrate of acute inflammatory cells was fund, grade 2 was defined. When neither ulceration nor acute inflammatory cells infiltrate were present, the grade 1 was given, when moderate to severe infiltrate of chronic inflammatory infiltrate was present. Grade 0 meant no or mild increases of chronic inflammatory cells and absence of acute inflammatory cells and ulceration.

3. Imaging Protocol of PET-MR Enterography

After a fasting period of at least 6 hours prior to the examination, patients received an intravenous administration of bodyweight-adapted [18F]-FDG (mean dose 178 ± 46 MBq) 60 minutes before PET/MR scan. For bowel distension 1500 ml of a solution containing 2.5% mannitol and 0.2% locust bean gum was ingested within 45 minutes prior to examination. Patients were placed in prone position. PET acquisition time per bed-position took 8 min. and 3 bed-positions were needed. PET images were reconstructed using a 3D (three-dimensional) Ordinary Poisson Ordered-Subset Expectation Maximation algorithm (3D OP-OSEM; 3 iterations, 21 subsets, voxel size 2.1x2.1x2.0 mm3, 3D Gaussian filter of 4.0 mm). The simultaneously acquired MR sequences were summarized in table 1.

Table 1 Technical details of MR parameters obtained in the PET-MR enterography protocol

**MR-Sequences**	**Plane**	**TE / TR (ms)**	**Matrix size**	**Slice thickness (mm)**	**Field of View (mm)**
**TrueFISP (a)**	coronal	1.49 / 666	288 x 288	3	400
**T2 HASTE fs (b)**	coronal	85 / 1800	256 x 288	5	400
**ce T1_3D VIBE fs (c)**	coronal	1.51 / 4.08	320 x 195	2	400
**ce T1_3D VIBE fs (d)**	axial	1,53/3,97	320 x 180	3.5	380
**EPI DWI (e)**	axial	86/ 1190	192 x 192	5	380

a) fast steady-state precession sequence (TrueFISP) without fat suppression.

b) fat-saturated T2-weighted (T2w) half-Fourier acquisition single-shot turbo spin echo (HASTE).

c) contrast-enhanced 3D T1w Volumetric Interpolated Breath Hold Examination (VIBE) with repetitive scans after i.v. injection of 0.2 ml/kg gadoteric acid (Dotarem®, Guerbet, Villepinte, France).

d) contrast-enhanced 3D T1w Volumetric Interpolated Breath Hold Examination (VIBE) with 180 seconds delay.

e) diffusion weighted echo planar sequence (EPI DWI, B-values: 0, 500 and 1000 s/mm^2).

4. Receiver operating characteristics curves of SUVmaxQuot in detecting endoscopically active and severe inflammation with and without purgatives.

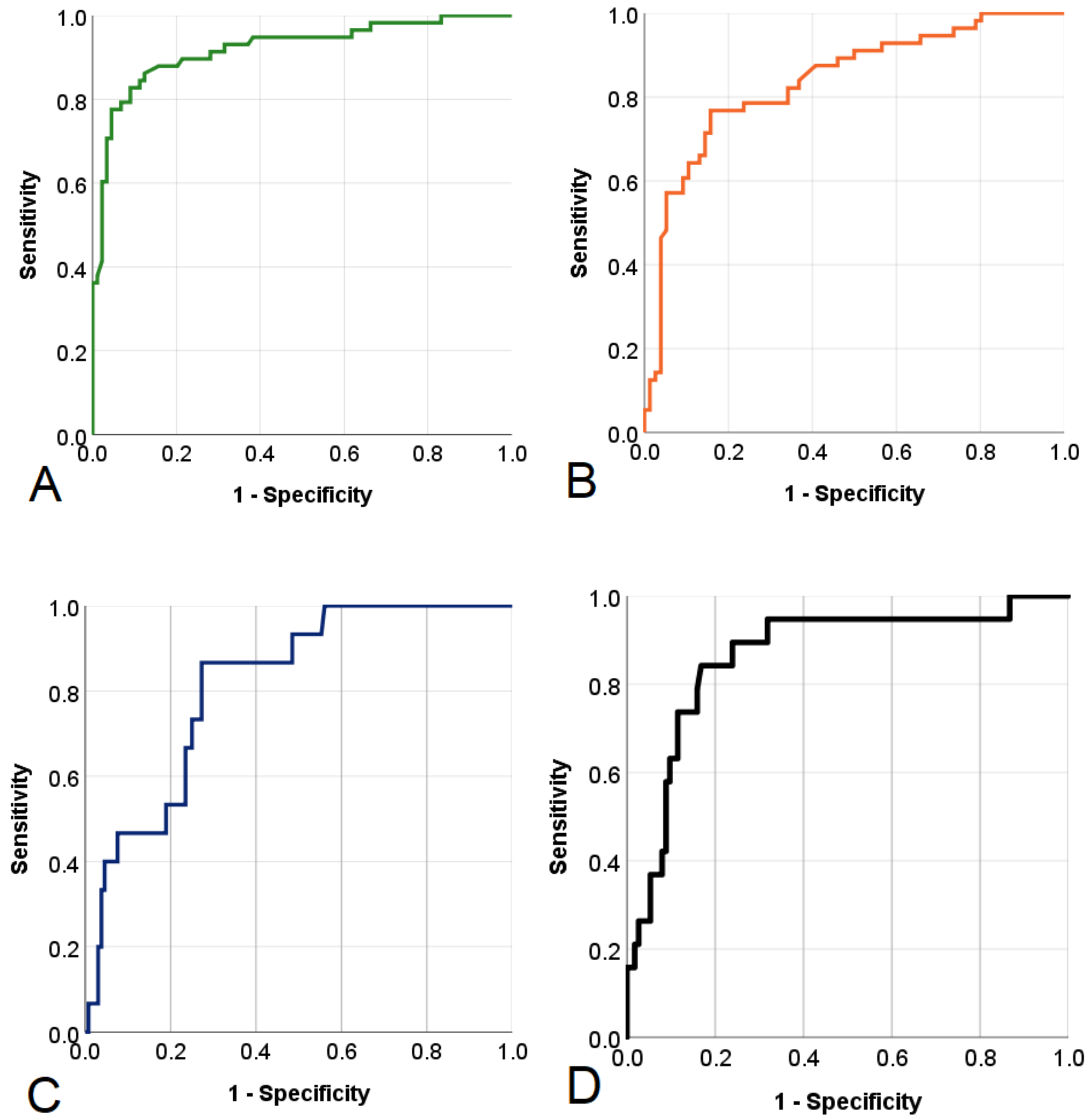

Figure A: ROC curve of SUVmaxQuot in detecting active inflammation in bowel segments without purgation (AUC = 0.921).

Figure B: ROC curve of SUVmaxQuot in detecting active inflammation in bowel segments with purgation (AUC = 0.836).

Figure C: ROC curve of SUVmaxQuot in detecting severe inflammation in bowel segments without purgation (AUC = 0.816).

Figure D: ROC curve of SUVmaxQuot in detecting severe inflammation in bowel segments with purgation (AUC = 0.863).

Note. AUC = Area under the curve; ROC = receiver operating characteristics
